# Laccase Immobilization Strategies for Application as a Cathode Catalyst in Microbial Fuel Cells for Azo Dye Decolourization

**DOI:** 10.3389/fmicb.2020.620075

**Published:** 2021-01-18

**Authors:** Priyadharshini Mani, V. T. Fidal, Taj Keshavarz, T. S. Chandra, Godfrey Kyazze

**Affiliations:** ^1^School of Life Sciences, University of Westminster, London, United Kingdom; ^2^Department of Biotechnology, Indian Institute of Technology (Madras), Chennai, India

**Keywords:** laccase, immobilization, acid orange 7, power production, decolourization

## Abstract

Enzymatic biocathodes have the potential to replace platinum as an expensive catalyst for the oxygen reduction reaction in microbial fuel cells (MFCs). However, enzymes are fragile and prone to loss of activity with time. This could be circumvented by using suitable immobilization techniques to maintain the activity and increase longevity of the enzyme. In the present study, laccase from *Trametes versicolor* was immobilized using three different approaches, i.e., crosslinking with electropolymerized polyaniline (PANI), entrapment in copper alginate beads (Cu-Alg), and encapsulation in Nafion micelles (Nafion), in the absence of redox mediators. These laccase systems were employed in cathode chambers of MFCs for decolourization of Acid orange 7 (AO7) dye. The biocatalyst in the anode chamber was *Shewanella oneidensis MR-1* in each case. The enzyme in the immobilized states was compared with freely suspended enzyme with respect to dye decolourization at the cathode, enzyme activity retention, power production, and reusability. PANI laccase showed the highest stability and activity, producing a power density of 38 ± 1.7 mW m^−2^ compared to 25.6 ± 2.1 mW m^−2^ for Nafion laccase, 14.7 ± 1.04 mW m^−2^ for Cu-Alg laccase, and 28 ± 0.98 mW m^−2^ for the freely suspended enzyme. There was 81% enzyme activity retained after 1 cycle (5 days) for PANI laccase compared to 69% for Nafion and 61.5% activity for Cu-alginate laccase and 23.8% activity retention for the freely suspended laccase compared to initial activity. The dye decolourization was highest for freely suspended enzyme with over 85% decolourization whereas for PANI it was 75.6%, Nafion 73%, and 81% Cu-alginate systems, respectively. All the immobilized laccase systems were reusable for two more cycles. The current study explores the potential of laccase immobilized biocathode for dye decolourization in a microbial fuel cell.

## Introduction

Microbial Fuel cells (MFCs) have been extensively explored for treatment of dye containing wastewater and concomitant energy production (Gomaa et al., 2017; Huang et al., 2017; Sonu et al., 2020). There are several studies reporting the use of enzymes and their fungal sources in MFCs for dye decolourization (Simões et al., 2019; Liu et al., 2020). Laccase is one such enzyme used in the textile industry for bleaching and in fuel cells for catalyzing the oxygen reduction reaction. Laccase is a multi-copper containing oxidase enzyme which is capable of one electron oxidation of other substrates and four electron reduction of O_2_ to H_2_O (Galhaup and Haltrich, 2001). Laccase has been employed in the cathode chamber of MFC for dye decolourization and catalysis of the oxygen reduction reaction (Savizi et al., 2012; Mani et al., 2019).

The use of enzymatic cathodes is however, limited by the short lifetime and stability of the enzymes in MFC systems due to factors such as pH gradients, salinity increases etc (Mani et al., 2017). These limitations could be overcome by immobilizing the enzymes to improve stability. Enzymes in the immobilized form are stable and resistant to environmental factors and the enzymes can be reused. When laccase was electropolymerized (120 U mg^−1^ of chitosan) on an electrode with chitosan and methylene blue and utilized in a MFC for dye decolourization, the MFC produced a maximum power density of 58.8 mWm^−2^ and a decolourization efficiency of 74% for Reactive Blue 221 dye after 120 h (Savizi et al., 2012). The different types of enzyme immobilization and their advantages and disadvantages are listed in Table 1.

**Table 1:** Types of enzyme immobilization, their advantages, and disadvantages.

**Types of immobilization**	**Description**	**Advantage**	**Disadvantage**
Adsorption	Enzymes are adhered to surface of carrier matrix through ionic, hydrophobic or van der Waals interaction (Jesionowski et al., 2014)	1. Relatively simple 2. Reduces conformational changes or denaturation of enzymes 3. Suitable for wide variety of carriers (Huang and Cheng, 2008)	1. Weak bonding (Cooney et al., 2008) 2. Exposed to microenvironment (pH, Temperature) 3. Depends on affinity between enzyme and carrier matrix
Covalent bonding (Cross-linking)	Enzyme is attached to the matrix by covalent bonds (Guisan, 2006).	1. Strong Bonding 2. No leakage 3. Higher stability (Romo-Sánchez et al., 2014)	1. Enzyme loading limited by matrix functional group density (Cooney et al., 2008) 2. Structural and conformational change 3. Diffusional limitation to the active site of the enzyme
Encapsulation	Enzyme is caged micelles of polymer having hydrophobic interior and hydrophilic exterior (Moehlenbrock and Minteer, 2017)	1. Retains native enzyme structure 2. Minimal enzyme requirement 3. No chemical modification	1. Not suitable for large substrates 2. Diffusional limitation (Cooney et al., 2008) 3. Microcapsules are sensitive to surrounding medium to like pH, ionic strength etc.
Entrapment	Enzyme is caged in a porous matrix by covalent or non-covalent bonds (Datta et al., 2013)	1. Retains native enzyme structure 2. Minimal enzyme requirement 3. No chemical modification	1. There polymer used in entrapment might be charged resulting in lower activity. 2. Difficult to control the pore size 3. Enzyme leaching 4. Diffusional barrier

Immobilizing enzymes on electrodes was found to provide direct electron transfer and higher power output in enzymatic biofuel cells (Cooney et al., 2008). Conducting polymers like Poly Aniline (PANI) can form an adhesive polymer on electrodes and conduct electrons (Tiwari et al., 2007). Another method of immobilizing enzymes is entrapment of the enzyme in beads. Laccase is a copper-dependent enzyme and immobilizing in copper alginate beads could retain more activity compared to other methods. Teerapatsakul and group have observed that the immobilization yield and enzyme activity was higher when CuSO_4_ was used as crosslinking solution compared to CaCl_2_ (Teerapatsakul et al., 2008).

A third method of immobilization involves encapsulation of laccase in Nafion polymer micelles formed by modifying the polymer with an alkyl ammonium salt such as tetrabutylammonium bromide (TBAB) (Meredith et al., 2012). The quaternary ammonium cations modify the Nafion to less acidic form by replacing the protons and counteracting the sulfonate groups. They also increase the size of micelles and channels which should result in favorable enzyme immobilization.

Platinum is the most commonly used cathode catalyst for high performance fuel cells. In recent years, because of the high cost of platinum, there has been a transition to PGM (Platinum Group Metals) free catalysts for oxygen reduction reactions with metal compounds impregnated with Nitrogen doped Carbon (N-C) serving as a good replacement for Pt. Transition metals such as Mn, Fe, Co, and Ni in their salt forms have been infused with precursor aminoantipyrine (AAPyr) and used as cathode catalysts in MFCs (Kodali et al., 2017).

In this study, laccase in the three immobilized states (Cross-linking, entrapment in beads, and micellar encapsulation) was compared with freely suspended enzyme with respect to dye decolourization, enzyme activity retention, power production, and reusability in the cathode of a microbial fuel cell. This study aimed to emphasize the effect of immobilization on laccase ability to perform as efficient cathode catalysts. The performance of the laccase electrode in terms of power generation only was also evaluated against platinum and chemical based platinum alternatives (Fe-N/C catalyst).

## Materials and Methods

### Chemicals

Laccase enzyme (13.6 U/mg) from *Trametes versicolor* was obtained from Sigma Aldrich. All chemicals were analytical grade and were purchased from Sigma. *Shewanella oneidensis MR1* strain 14063 was purchased from NCIMB (UK). The Fe-N/C catalyst was obtained from Dr. J. Masa from Ruhr-University Bochum in Germany.

### Laccase Immobilization

#### Polyaniline Laccase

Polyaniline (PANI) immobilization was carried out by electropolymerization of 0.1 M Aniline in 1 M Sulphuric acid with carbon fiber (2.5 cm^2^) as working electrode, titanium wire as counter electrode and Ag/AgCl as reference using Keysight B2900A potentiostat. A current density of 4.5 mA cm^−2^ for 50 sec was used for electropolymerization of aniline onto bare carbon electrodes. After electropolymerization, PANI was functionalised using 1.25% glutaraldehyde at 37°C for 15 min. This was followed by the addition of laccase enzyme 1 U ml^−1^ (60 Units) to the solution for cross linking for 15 min. Enzyme assay of the laccase solution was carried out before and after cross linking to get an estimate of amount of laccase immobilized (Mani, 2019).

#### Copper Alginate Beads

The Cu-Alginate immobilization procedure was adapted from Teerapatsakul et al. (2008). A 3% w/v sodium alginate was dissolved in 40 ml of water. A 1 U ml^−1^ (60 Units) laccase was added to the alginate solution. The above mixture was passed through a 21-gauge syringe into 0.15 M cross-linker copper sulfate solution. The beads were allowed to rest for 45 min after which they were washed with and incubated in acetate buffer. Enzyme assay was performed on the laccase immobilized Cu-Alg beads and the remaining cross-linking solution to determine the immobilization yield.

#### Nafion Micelles Preparation

The salt modified Nafion micelles were prepared according to the method developed by Meredith et al. (2012). Two ml of 5% w/v Nafion solution (Sigma) was added to 78.3 mg of TBAB (tetrabutylammonium bromide) and vortexed at 1,500 rpm for 10 min. The solution was poured in a weighing boat and the solvent was allowed to evaporate. After 18 h a yellow transparent film was formed at the bottom of a weighing tray. The tray was then filled with 18 MΩ deionised (DI) water and soaked for 24 h to remove the excess alkyl ammonium bromide salts and HBr. The water was removed, and the polymer rinsed with DI water and allowed to dry. The resulting dry film was suspended in 2 ml ethanol.

#### Immobilization of Laccase in Nafion Micelles

Laccase was dissolved in 10 ml of acetate buffer (pH 4.5) to give a concentration of 1 U ml^−1^(60 Units). 1 ml of the ethanol-polymer suspension was added to 2 ml of the enzyme solution and vortexed. The resultant mixture was poured onto an electrode and the solvent allowed to evaporate, thus forming a film on the electrode surface.

### Platinum and Fe–N/C Electrode Preparation

The cathode contained a Pt catalyst layer with a Pt loading of 0.5 mg cm^−2^. Pt powder for the cathode was mixed with carbon black powder (Sigma Aldrich, UK) for a 10% (w/w) mixture. This mixture was suspended in Nafion solution (Sigma Aldrich) and the suspension was applied as a uniform coating on the cathode electrodes using a paint brush. The same approach was used for Fe—N/C catalyst electrode preparation.

### Operation of the Microbial Fuel Cell

The MFC used in the study was the 'H'-type reactor with a working volume of 200 ml in each chamber. The electrodes were constructed from carbon fiber (non-woven) with a surface area of 25 cm^2^. Cation exchange membrane CMI7000 ion exchange membrane was soaked in 5% NaCl for 12 h prior to use.

### Anode Chamber Composition

The composition in the anode chamber was the same for all the reactors. The anolyte consisted of minimal salts medium containing (per liter): 0.46 g NH_4_Cl, 0.22 g (NH)_2_SO_4_, 0.117 g MgSO_4_, 7.7 g Na_2_HPO_4._7H_2_O, 2.87 g NaH_2_PO_4_ along with 1% (v/v) trace minerals as described by Marsili et al. (2008) and 1% (v/v) vitamin mix as described by Wolin et al. (1963). The carbon source was pyruvate at a concentration of 1 g L^−1^ and casein hydrolysate was added at 500 mg L^−1^. The pH of the anolyte was initially adjusted to seven. The anode and cathode were connected to a resistor of 2 kΩ. The anode was inoculated with 10% v/v *S. oneidensis* MR-1 culture previously grown in Luria Bertani broth to an OD of 0.6. The anode chamber was sparged for 10 min with nitrogen gas to remove any dissolved oxygen and maintain an anaerobic environment.

### Cathode Chamber Composition

The cathode chamber consisted of commercial laccase (Sigma–Aldrich) from *Trametes versicolor* (13.6 U mg^−1^) in 100 mM sodium acetate buffer solution (pH 4.5). Free laccase was added at 0.3 U/ml (60 Units/ 200 ml) in the cathode chamber. A total of 60 Units was maintained initially for all laccase immobilized systems.

### Experimental Design

Four systems were setup with laccase in the cathode chamber and one without enzyme: System 1 with Polyaniline crosslinked to laccase electrode referred to as “PANI laccase” to reduce ohmic loss; System 2 with laccase entrapped in copper alginate beads referred to as “Cu-Alg Laccase” to reduce enzyme denaturation; System 3 with laccase freely suspended in the buffer referred to as “Free laccase;” System 4 with laccase immobilized in Nafion micelles is referred to as “Nafion Laccase” to maintain activity and System 5 referred to as “Control” which consisted of dye and buffer in the absence of laccase. The immobilized and free enzymes were suspended in 200 ml of 100 mM acetate buffer (pH 4.5) with 100 mg L^−1^ of Acid Orange 7 dye. The cathode chamber was maintained in aerobic conditions by supplying air through an air stone at a rate of 200 ml air per min. Experiments were conducted at a temperature of 30°C. For platinum comparison System 1 with platinum coated electrode is referred as “Platinum” and System 2 with Fe-N/C coated electrode is referred to as “Fe-N/C.” The immobilized enzymes, platinum and Fe-N/C electrode were suspended in 200 ml of 100 mM acetate buffer (pH 4.5) in the absence of the dye. The MFC systems were connected across 2000 Ω resistor. The experiments were carried out in batch mode with one cycle in this study representing 5 days.

### Analytical Procedures

#### Structural and Functional Characterization of the Electrodes

The morphology of the immobilized laccase was studied using Inspect-F scanning electron microscopy (SEM) equipped with EDAX at an accelerating voltage of 30 kV. The presence of PANI and Nafion functional groups was detected using Perkin Elmer Spectrum Two FTIR-ATR Spectrometer with a plain carbon electrode as the background.

#### Electrochemical Analysis of Laccase Crosslinked With PANI and Laccase in Nafion Micelles

The cyclic voltammetry (CV) measurements for activity of laccase was performed in a three-electrode system with the working electrode as the PANI laccase/Nafion laccase electrode, platinum as the counter, and Ag/AgCl as reference electrode. Since the enzyme was immobilized on the electrode, ABTS assay did not serve as an accurate method for enzyme activity, as the enzyme leached into the solution also accounted for the laccase activity rather than the enzyme on the electrode alone. The CV was carried out in pH 4.5 acetate buffer (100 mM) using a potentiostat Keysight B2900A by cycling between potential of −1–1.5 V at a scan rate of 20 mV s^−1^.

#### Acid Orange 7 Dye Decolourization

The concentration of AO7 in the cathode for laccase systems was measured at various time intervals using a UV-visible spectrophotometer at a wavelength of 484 nm which is the maximum absorption wavelength for the dye. The decolourization efficiency was calculated by

$$\begin{array}{l} \text{DE}\left(\text{\%}\right)=\frac{\text{A}\text{o}-\;{\text{A}}_{\text{t}\;}}{\text{A}\text{o}}\;\times \;100 \end{array}$$

A_o_ and A_t_ are the absorbance units at the initial and each time point, respectively. A time series is plotted for the absorbance values measured.

#### Electrochemical Analysis

The electric potential across the system was recorded every 10 min using a data acquisition system Picolog (Pico Technology, UK).

Power and current density were normalized to the surface area of the anode electrode. To carry out polarization tests, each MFC unit was connected to various external resistances ranging from 10 Ω to 1 MΩ and the potential measured using a multimeter. Internal (ohmic) resistance was calculated from the slope of the linear portion of the polarization (V-I) curve.

#### Coulombic Efficiency

The CE was calculated as follows (Logan et al., 2006):

$$\begin{array}{l} \text{CE}(\%)=\frac{M\;\int_{0}^{t}I\;dt}{b*F*V_{anode}*\Delta COD} \end{array}$$

where M is the molecular weight of oxygen (32), I is current over a time period (A), b number of electrons exchanged per mole of oxygen, F is Faraday constant (96485C mol^−1^), V_anode_ is working volume of anode, and COD is change in COD over time (g L^−1^).

#### Enzyme Activity

The activity of free laccase and coper alginate immobilized laccase was measured using ABTS [2,2′-azino-bis(3-ethylbenzothiazoline-6-sulphonic acid)] as a substrate. A solution of 2 ml acetate buffer (100 mM, pH 4.5), 0.1 ml ABTS (0.5 mM), and 0.1 ml of enzyme was used for freely suspended enzyme and 300 mg for Cu-alginate beads. The oxidation of ABTS by laccase was measured by a UV spectrophotometer at 420 nm (Bakhshian et al., 2011). The enzyme activity unit (U) was defined as the amount of enzyme required to oxidize 1.0 μmol ABTS min^−1^ at 25°C (Eggert et al., 1996).

#### Immobilization Yield

The immobilization yield of the enzyme was calculated by:

$$\begin{array}{l} \text{Immobilization yield}\;\left(\text{\%}\right)=\;\frac{\text{Amount of Enzyme immobilized}}{\text{Total Enzyme used in immobilization}}\;\text{x}\;100 \end{array}$$

The amount of enzyme immobilized was calculated by the ABTS enzyme assay.

### Statistical Analysis

All experimental data indicated in the text and graphs are the means of triplicate experiments unless otherwise stated. The error bars in the graphs and ± values in the text represent the standard deviation of the mean (SD).

## Results and Discussion

### Characterization of Immobilized Laccase Biocathode Systems

The immobilized laccase biocathodes were analyzed for their functional, morphological, and electrochemical characteristics using FTIR, SEM, and cyclic voltammetry, respectively.

#### Functional Analysis of Laccase Biocathodes

Functional analysis was performed for polymer-based laccase immobilized biocathodes viz. PANI-laccase and Nafion-laccase. As both the immobilizations were multi-step procedures it was necessary to understand the modifications in the polymer during each step and the robustness of the laccase on immobilization.

**Figure 1:**
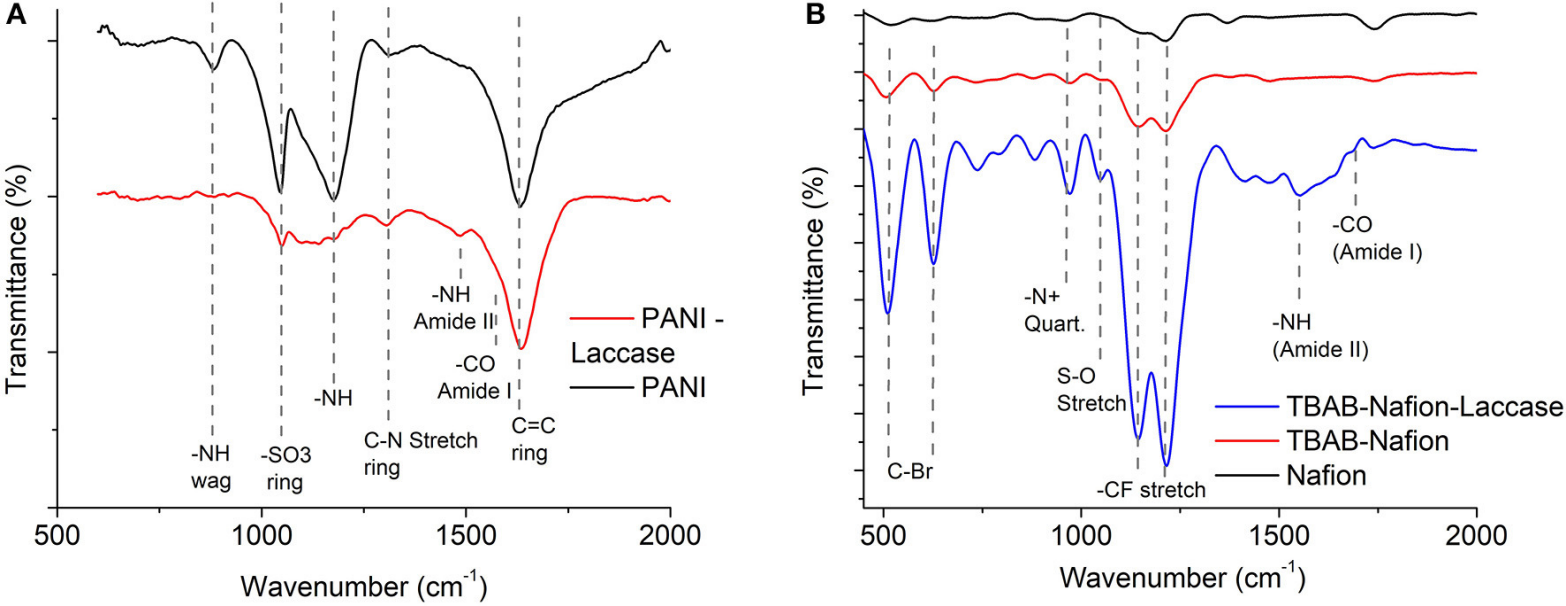
FTIR spectra indicating the presence of functional groups of polyaniline, nafion, and immobilized laccase **(A)** PANI laccase **(B)** Nafion laccase.

FTIR was carried out to confirm the presence of PANI functionalization on the electrode (Figure 1A). The 1314 cm^−1^ is typical of PANI (emeraldine base) attributed to C-N stretch vibration of the quinoid ring. The peak at 1175 cm^−1^ indicates the vibration mode of –NH^+^ of the charged polymer. After the glutaraldehyde cross linking, this mode disappears due to crosslinking with laccase. This mode in PANI is responsible for the delocalized electron and hence the conductivity. The peak at 882 cm^−1^ corresponds to the N-H wag of the 1° and 2° amine which disappears on cross-linking with the enzyme (Figure 1A). Another peak characteristic of the PANI deposited in sulphuric acid is observed at 1047 cm^−1^ which is due to the sulphonation of the aniline ring due to the substitution of the ${SO}_{3}^{-}$ in place of ${NH}_{3}^{+}$ (Figure 1A). The strong peak observed at 1627 cm^−1^ is due to the C=C in vibration within the ring (Stejskal and Gilbert, 2006).

The Nafion functional group showed a CF stretch at 1134 cm^−1^ and 1213 cm^−1^ which is characteristic of tetrafluoroethylene backbone (Figure 1B). The mild peak at 1048 cm^−1^ indicates the sulfonated terminal of the tetrafluoroethylene chain (Kunimatsu et al., 2010). On functionalization with TBAB, a peak appeared at 978 cm^−1^ which indicated the presence of 40 N+ embedded within the polymer (Figure 1B) (Hu et al., 2016). On laccase immobilization, the peak intensity increased, which might be due to the catalysis and higher vibrational modes achieved due to the charging of the polymer. The laccase was characterized by the peaks at 1559 and 1959 cm^−1^ which indicates the amine and carboxylic moiety of its amino acids (Figure 1B). For both PANI and Nafion immobilization, laccase was characterized by the presence of Amide I (1600–1690 cm^−1^) and Amide II (1480–1575 cm^−1^) for -CO and -NH stretch, respectively (Kong and Yu, 2007).

#### Morphological Analysis of the PANI/PANI-Laccase Biocathode

Morphological analysis was performed for PANI laccase, Cu-Alg laccase, and Nafion laccase biocathode systems. The main significance of this study was to understand the porosity of the electrode and structural changes in the polymer on immobilization of the laccase.

**Figure 2:**
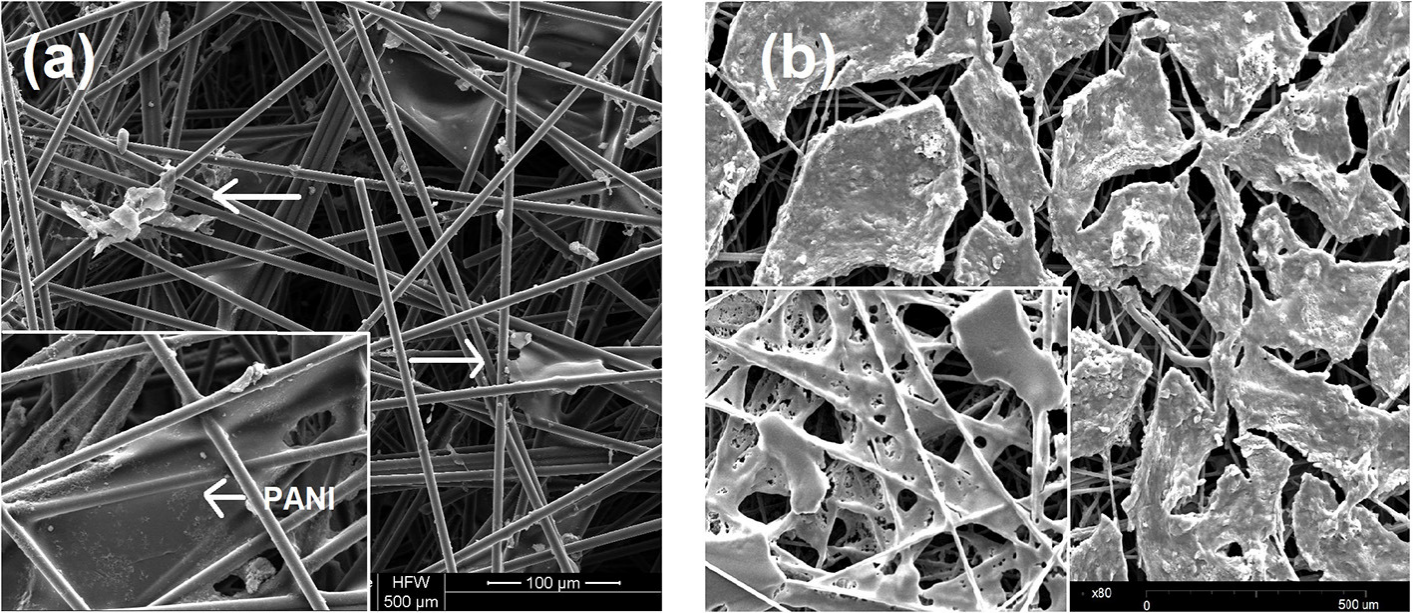
SEM image of **(a)** PANI laccase. Arrows indicate laccase enzyme, Inset (left): PANI membrane **(b)** Nafion/TBAB/Laccase, Inset-Nafion/TBAB.

SEM images reveal the PANI fibers on the carbon electrode and the immobilized laccase (Figure 2a-Inset). PANI appears as a polymer sheath formed over the carbon fibers (Figure 2a-Inset). It is deposited primarily at the tight fibers of carbon due to higher charge density, with dimensions in the range of 30 × 50 μM. Crosslinking causes slight disruption of the membrane with the globular structure more prominent after laccase immobilization (Figure 2a).

TBAB modified Nafion was found to coalesce to form a film on the electrode surface (Figure 2b-Inset). Unlike PANI film, the Nafion membrane is evenly distributed over the carbon filaments. The porosity of the electrode was found to be decreased on the film formation, which might affect the charge density. A gelation of the Nafion polymer is seen on addition of TBAB. In the presence of laccase, the film appears to be a thick layer of membrane compared to bare Nafion/TBAB (Figure 2b). Unlike PANI-Laccase, Nafion-laccase film was seen to be restricted to the surface of the electrode. In addition, the aggregate size of the enzyme-polymer was larger in size as compared to the PANI-laccase.

#### Electrochemical Analysis of PANI and Nafion-Laccase Electrodes

The electrochemical analysis of the immobilized laccase biocathodes were limited to the PANI-laccase and Nafion-laccase biosystems because of the conductivity of the polymer used.

**Figure 3:**
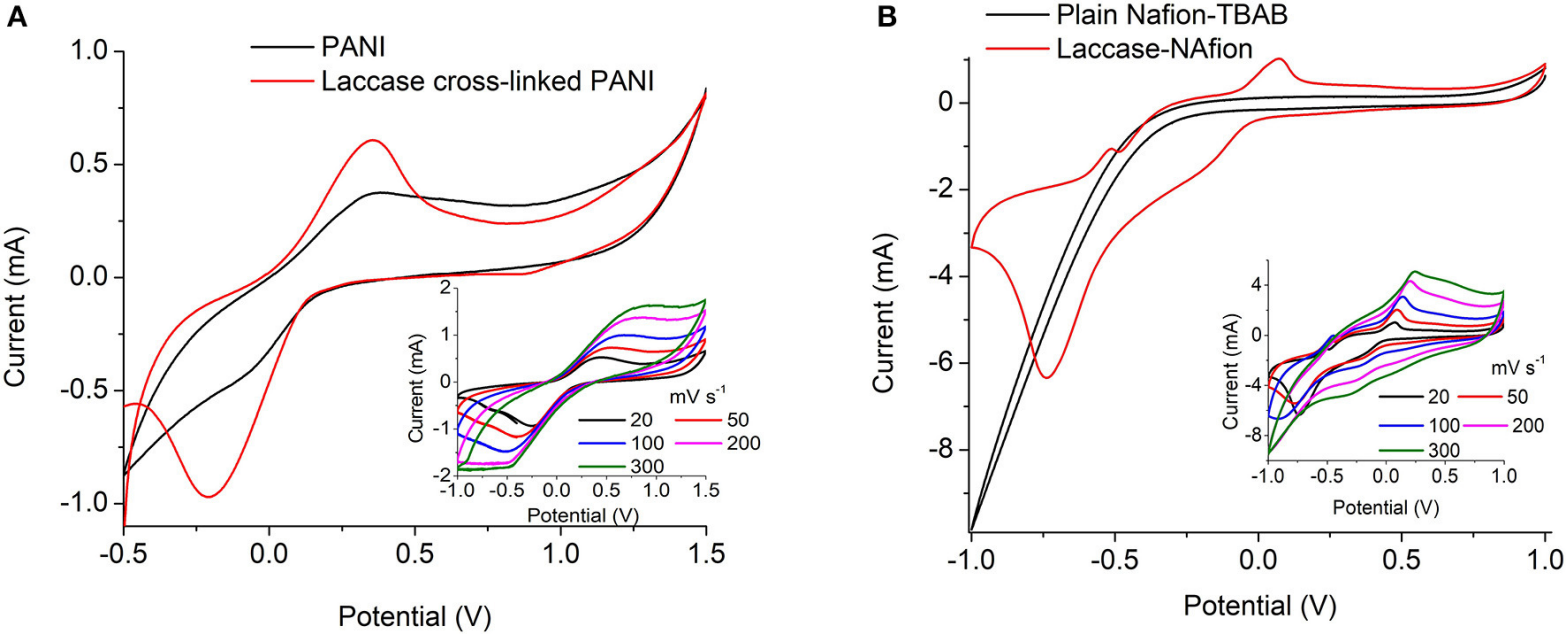
Cyclic voltammetry at 20 mV s^−1^ of **(A)** PANI and PANI cross-linked with laccase **(B)** Nafion/TBAB and Nafion/TBAB/laccase system. Insets: Immobilized laccase at different scan rates.

The redox property of the PANI-Laccase biocathode was analyzed by cyclic voltammetry. An oxidation peak at 0.2 V indicates the presence of polyaniline on the electrode surface (Figure 3A).

PANI did not display the multiple peaks usually observed in strong acids, as the electrolyte used in this study was a weak acetate buffer. Polyaniline is easily oxidized in less acidic solutions as pH increases. The electrochemical behavior of polyaniline is dependent on many parameters e.g., the choice of material and the surface area of the electrodes, composition of the electrolyte, pH and temperature etc. (Song and Choi, 2013). On cross-linking with laccase an additional peak was observed at −0.32 V which might indicate oxygen reduction reaction (Le Goff et al., 2015). With increasing scan rate the rise in cathodic peak current was proportional to the square root of scan rate, indicating an oxygen diffusion limited process (Figure 3A-Inset). Thus, the laccase catalytic activity was preserved on cross-linking with PANI (Figure 3A).

Nafion polymer alone did not show any characteristic peaks due to absence of the characteristic redox moiety. The increase in oxidation and reduction current in laccase modified electrode indicates the presence of immobilized enzyme on the surface of the electrode. Similar shifts in CV current was also observed by Luo et al. (2010) for Nafion/ABTS/laccase electrodes. In presence of laccase the ORR takes place on the surface and the reduction peak appears at −0.6 V limited by oxygen diffusion (Figure 3B-Inset). Nafion-laccase showed an overpotential as compared to the PANI laccase, which might be due to poor electron conductivity of the Nafion (Figure 3B).

### Power Generation From MFCs Utilizing the Immobilized Laccase Electrodes

**Figure 4:**
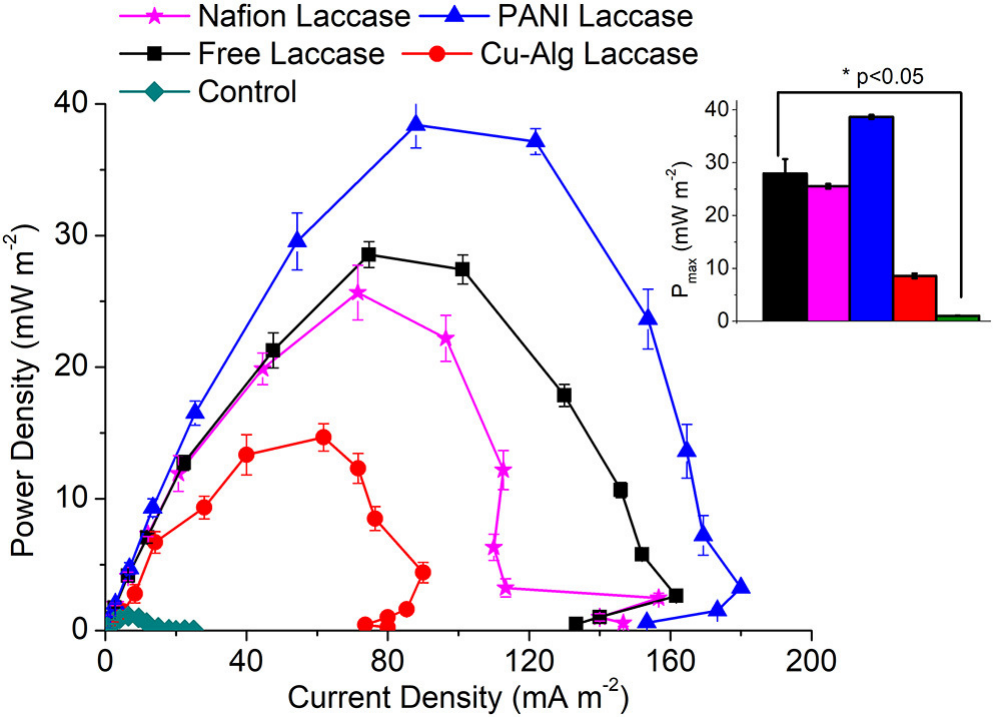
Comparison of P_max_ (maximum power density) for the different Laccase based biocathode systems. Inset: One-way Anova of maximum power density at day 5 for each MFC systems.

The maximum voltage of 480 ± 20 mV was recorded across 2 kΩ in the MFC with PANI laccase followed by freely suspended laccase (420 ± 14 mV), Nafion-laccase at 405 ± 30 mV, and Cu-Alginate laccase at 350 ± 25 mV. The higher voltage of PANI MFCs compared to those with freely suspended laccase was probably due to the decreased proximity between the catalytic sites and the electrode, thus decreasing the ohmic and mass transfer resistance. Moreover, PANI is a conducting polymer, it decreases the charge transfer resistance of the electrode thus permitting the easy electron transfer. Although Nafion is also a conducting polymer, it is known to be an ionic conductor rather than an electron conductor (Heitner-Wirguin, 1996). In this study, laccase embedded in the Nafion without any mediators was less efficient in transferring electrons from the electrode to the enzyme compared to PANI. The low voltage of Cu-Alg laccase system was probably due to the high diffusion barrier imposed by beads to both oxygen and electron transfer from the electrode. This agreed with the maximum power density which was 38 ± 1.7 mWm^−2^ for MFCs with PANI and 28 ± 0.98 mWm^−2^ for freely suspended laccase, 25.6 ± 2.08 mW m^−2^ for Nafion and 14.7 ± 1.04 mW m^−2^ for laccase entrapped in beads (Figure 4). A maximum power density of only 6.5 mW m^−2^ was observed by Schaetzle et al. (2009) when laccase was immobilized in hydrogels due to the reduced electron transfer of the enzyme hydrogels (Schaetzle et al., 2009). Thus, it is evident that the distance between the enzyme and the electrode is critical in achieving good oxygen reduction and higher power output. PANI, Nafion and freely suspended enzyme have better contact with the electrode compared to the beads. The OCV for PANI laccase reached up to 900 ± 35 mV while for free laccase it was 700 ± 20 mV, 640 ± 48 mV for Nafion, and 500 ± 32 mV for laccase in Cu-alginate beads. The internal resistance for MFCs with PANI was 1.4 ± 0.15 kΩ which was the lowest compared to 2 ± 0.12 kΩ for free laccase and 7.5 ± 1 kΩ for the beads which was directly related to the above factors of ohmic and diffusion barrier. The internal resistance for nafion laccase was 5.3 ± 0.5 kΩ which might account for the low power output compared to PANI laccase. The coulombic efficiency was quite low for all systems with PANI-laccase highest at 4.65 ± 0.18%, Nafion with 4.23 ± 0.45%, Free laccase 3.83 ± 0.112%, and the lowest was Cu-Alg with 2.97 ± 0.16%. In addition, PANI laccase electrodes were reusable for up to three cycles with the power and activity decreasing each cycle. Nafion and Cu-Alg laccase were reusable for two more cycles (Data in Supplementary Tables 1, 2).

### Dye Decolourization in MFCs Utilizing the Laccase Biocathodes

**Figure 5:**
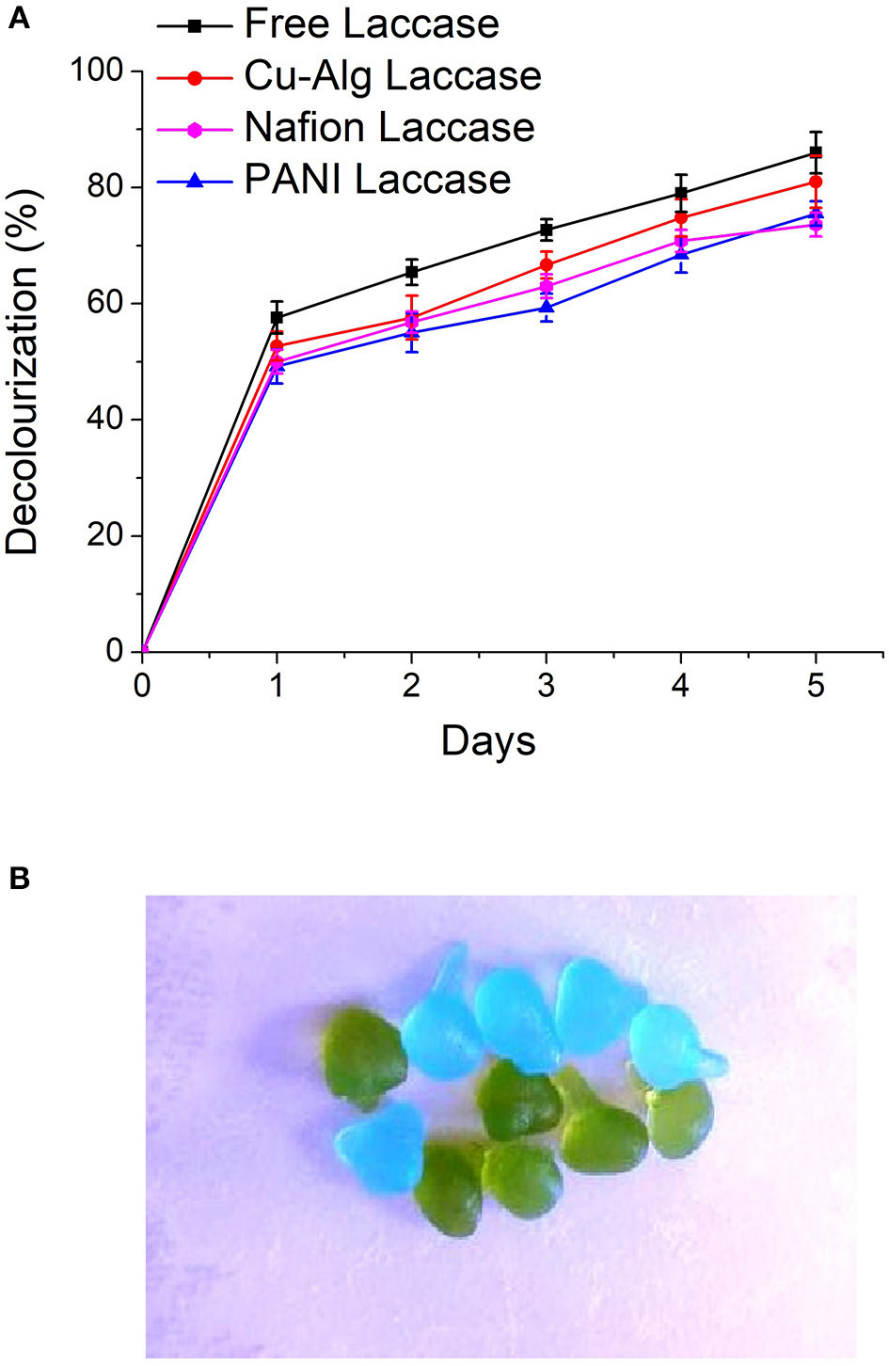
**(A)** Comparison of decolourization rates of AO7 in the different MFC systems **(B)** Dye adsorption in copper alginate beads. Blue–Initial bead color, Green–On adsorption of dye.

There was 85 ± 3.5% decolourization by MFC with enzyme in the freely suspended form compared to 75.6 ±2.1% for PANI laccase and 73 ± 2.04% Nafion laccase over a period of 5 days (Figure 5A). The decolourization in MFCs with Cu-Alg beads laccase was 81 ± 4.47%. There was >50% decolourization in the first 24 h for free and Cu-Alg laccase. Zille et al. (2003) inferred that on immobilization the protein becomes restricted to interact with the dye. Freely suspended laccase has the freedom of movement to interact with the dye and bring about better decolourization in this study. Same trend was obtained by Savizi et al., where freely suspended laccase enzyme decolourized 77% of RB 221 dye compared to 70% in immobilized laccase (Savizi et al., 2012). In addition, the amount of enzyme cross-linked on the PANI laccase was lower than the case of freely suspended enzyme due to the functional group density limitation of glutaraldehyde which also contributes to lower decolourization. Similarly, for Nafion-laccase limitation of the dye movement to the active site of the enzyme might have resulted in lower decolourization compared to free laccase and Cu-Alg laccase. The initial rapid decolourization is due to the high enzyme activity initially; the rate of decolourization decreases gradually as the enzyme activity decreases as shown in the enzyme activity graphs (Figure 7). The dye decolourization by laccase is probably due to asymmetric cleavage of the azo-bonds and subsequent ring cleavage to form simple aromatic compounds (Mani et al., 2019).

There was significant amount of dye adsorbed on the alginate beads which indicates that part of decolourization is due to adsorption (Figure 5B). Control beads without laccase showed 8 ± 0.5% decolourization of the dye.

Similar results were observed by Daâssi et al. (2014) where 34 and 24% of dyes Reactive Black and Lanset Gray, respectively was adsorbed on calcium alginate beads with laccase (Daâssi et al., 2014). The laccase beads were reusable for two more cycles with the decolourization decreasing gradually each cycle (69 and 57%). There was no further decolourization after 120 h in any systems. This decolourization pattern was also observed by (Russo et al., 2008) and they have concluded it might be due to the inhibition of laccase by the products of the dye.

### Enzyme Activity of the Laccase Biocathodes in MFCs

The immobilization yield of the laccase immobilized systems was obtained by comparing the activity of laccase prior to and after the immobilization. The immobilization efficiency was highest in Cu-Alg laccase with 73 ± 8% yield followed by Nafion and PANI with 57 ± 2 and 38 ± 1%, respectively.

**Figure 6:**
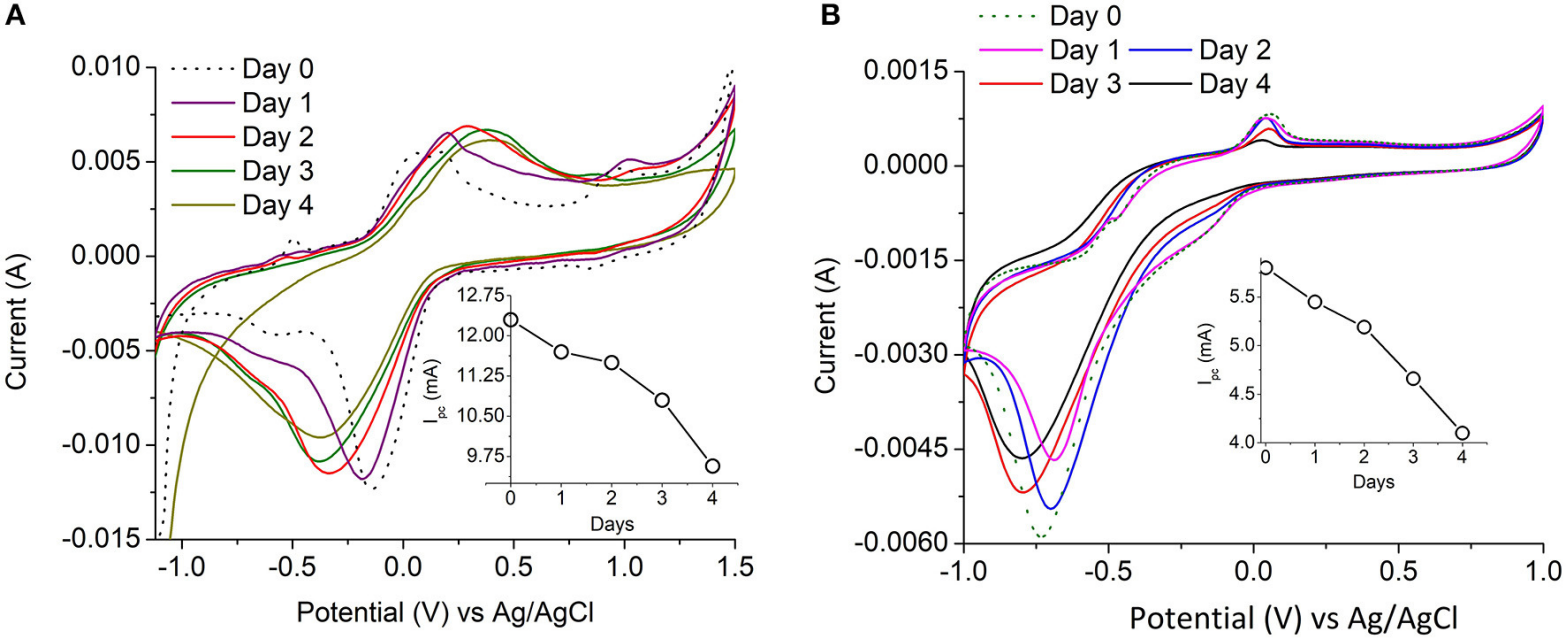
Laccase electrochemical activity with time **(A)** PANI-Laccase **(B)** Nafion-Laccase. Inset: Cathodic peak current vs. time at 20 mV s^−1^.

The enzyme activity was determined by ABTS assay for freely suspended laccase and for Cu-Alginate laccase. The relative decrease in activity for PANI-laccase and Nafion laccase on the electrodes were measured through cyclic voltammetry by comparing the cathodic peak current (Ipc) each day to the initial peak current (Figure 6). Cyclic Voltammetry of PANI laccase/ Nafion-Laccase indicated a decrease in Ipc with the number of days. (Figure 6-Inset).

**Figure 7:**
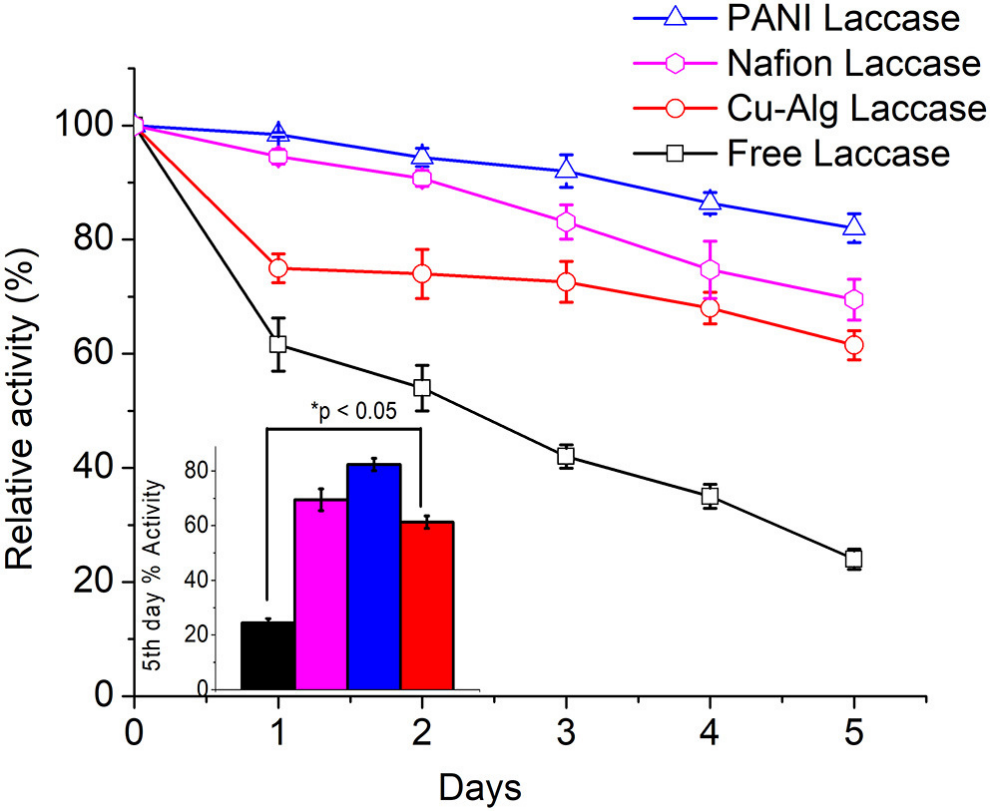
Comparison of relative laccase activity over time for each immobilized laccase system. Inset: One-way Anova of percentage activity at day 5 for each system.

The relative percentage decrease in enzyme activity for each electrode compared to their initial activity is shown in Figure 7. PANI Laccase retained 81 ± 2.5% activity after one MFC cycle (5 days), while freely suspended enzyme retained only 23.8 ± 1.8% activity after the first MFC cycle (5 days). The rate of enzyme deactivation was highest for freely suspended laccase with about 15.2 ± 0.12% decrease in relative enzyme activity per day. The enzyme activity was also observed to be decreasing in Nafion-laccase with loss of activity at the rate of 6 ± 0.39% per day in Nafion laccase compared to PANI laccase with just 3.8 ± 0.6% per day. There was >69 ± 3.5% enzyme activity retained in Nafion-laccase after one cycle (5 days). The laccase activity was five times higher when immobilized with Nafion-TBAB compared to plain Nafion. Similar results were observed by Meredith et al. (2012) for certain enzymes immobilized with Nafion modified with TBAB. The laccase entrapped in Cu-alginate beads had an initial burst release of 25 ± 0.8% within the 24 h of immobilization in the catholyte of MFC; following this, per day 4 ± 0.6% for Cu-Alg Beads with retention of 61.5 ± 2.5% after 5 days. The burst release might be due to repulsion between the negatively charged alginate (−29 mV) and laccase (−6 mV) at pH of 4.5 as observed with the zeta potential. Nafion polymer laccase possibly retained better activity compared to Cu-Alg due to lower leaching of the enzyme and well-protected microenvironment in the polymer micelle. Apart from Nafion, there have been work carried out with immobilization of laccase in other ionic liquids; the immobilization efficiency was high, but the voltage and power in MFC was much lower than the Nafion used in this study (Haj Kacem et al., 2018).

From the Table 2, PANI laccase shows the best performance of power density normalized with amount of enzyme immobilized and the amount of dye decolourized.

**Table 2:** Summary of the measured parameters for each system and the normalized power output.

**Laccase immobilization methods**	**Max. power density (mW m^**−2**^)**	**Dye decolourization (%)**	**Relative enzyme activity after 1 cycle (%)**	**Coulombic efficiency (%)**	Power per unit of enzyme per mg of dye decolourized (mW m^**−2**^ mg^**−1**^ U^**−1**^)^**^
PANI Lac	38 ± 1.7	75.6 ± 2.1	81 ± 2.5	4.65 ± 0.18	0.11 ± 0.003
Nafion Lac	25.6 ± 2.08	73 ± 2	69 ± 3.5	4.23 ± 0.45	0.05 ± 0.006
Cu-Alg Lac	14.7 ± 1.04	81 ± 4.47	61.5 ± 2.5	2.97 ± 0.16	0.02 ± 0.001
Free Lac	28 ± 0.98	85 ± 3.5	23.8 ± 1.8	3.83 ± 0.112	0.03 ± 0.0009

** *Power density (mW m^−2^) normalized with the amount of enzyme immobilized (U) with the amount of dye decolourized (mg) by each laccase immobilized systems*.

### Comparison of the Performance of Laccase Biocathode Systems With the Conventional Pt and Fe Impregnated Catalyst (Fe-N/C) in MFCs

**Figure 8:**
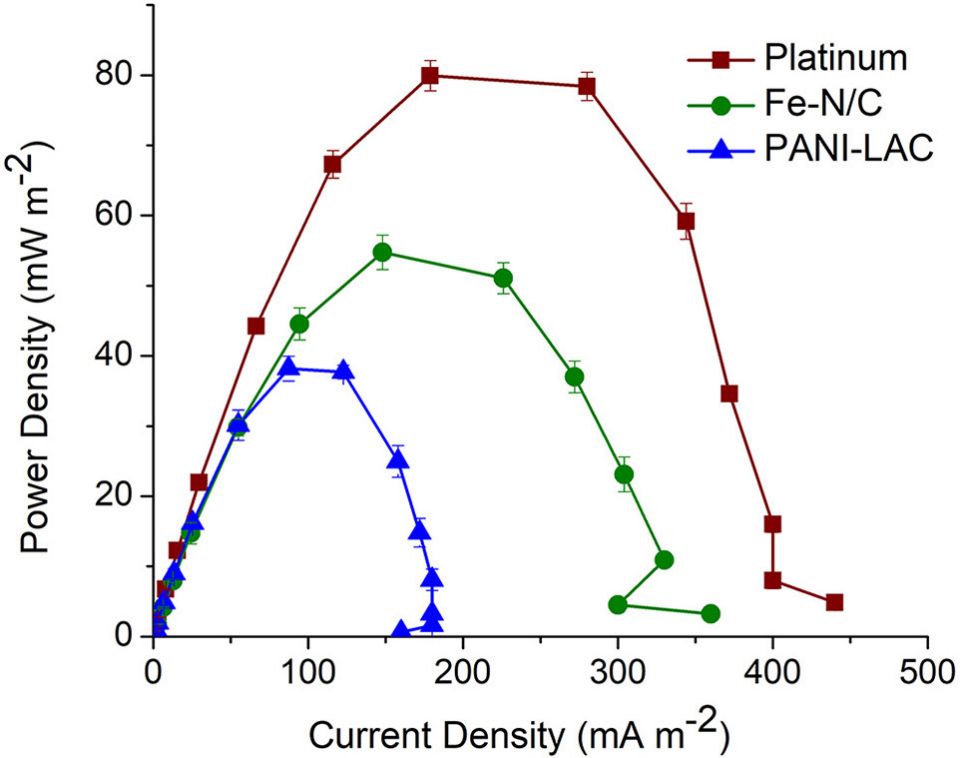
Comparison of maximum power production for Pt catalyst, Fe-N/C, and PANI-Laccase.

The laccase biocathode MFC systems above were compared with the traditional Pt and Fe impregnated N-doped carbon catalyst with regards to power generation in *Shewanella oneidensis*-based MFCs. Pt and Fe-N/C produced a power density of 80 ± 0.5 and 54.4 ± 0.84 mW m^−2^, respectively (Figure 8). The highest power density produced by laccase biocathode (PANI-Laccase) (38 ± 1.7 mW m^−2^) was much lower than that of Pt (80 ± 0.5 mW m^−2^, Figure 8), but factors such as cost of the enzyme and its concomitant dye decolourization rates serve as a major advantage. The cost of platinum is 2.5 times higher than that of laccase enzyme. one gm of platinum costs £198 (Sigma Aldrich) compared to laccase at £70/gm (Sigma Aldrich). Enzyme loading in our study is much less compared to other studies (Teerapatsakul et al., 2008; Savizi et al., 2012). The normalized power output for platinum was 0.04 and 0.07 mW/£ for laccase. Laccase therefore has 1.75 times higher power output per pound compared to platinum. The use of platinum electrodes in wastewater treatment can result in biofouling of the electrode and reduced power density (An et al., 2011). Choice of enzyme or platinum need not consider cost alone; other factors such as stability and reusability of catalyst, possible leaching of catalyst in the waste stream being treated, as well as effect of environmental conditions on catalyst performance should also be considered. Enzymes require optimized conditions for their performance such as stable pH, temperature etc. but have the versatility of being engineered for better performance.

## Conclusion

In this study, different methods of immobilization of laccase i.e., cross-linking with polyaniline (PANI), entrapment in Cu alginate beads and encapsulation in Nafion micelles, were investigated with regards to their application as biocathodes in Shewanella based MFC for purposes of azo dye decolourization. Laccase cross-linked with PANI served as an effective system in striking the right balance between maintenance of enzyme activity, dye decolourization efficiency, and power output. Many studies have used large enzyme loadings of 500–2000 U ml^−1^ and mediators for achieving simultaneous dye decolourization and high-power output in MFC. In this study, we have utilized much less enzyme loadings (maximum 0.3 U ml^−1^) in the absence of mediators to bring about decolourization of dyes and produce a good power output. The unstable nature of biological cathodes to wastewater is the major drawback for its efficiency in microbial fuel cells. Laccase has the versatility of being engineered for immobilization to extend their active lifetimes. As a result, with the advent of protein engineering laccase holds potential to be an excellent catalyst for oxygen reduction reactions to provide the comparable efficiency to that of metal catalysts.

## Data Availability Statement

The original contributions presented in the study are included in the article/Supplementary Materials, further inquiries can be directed to the corresponding authors.

## Author Contributions

GK designed the experiments and proof read the manuscript. PM and VF conducted the experiments and contributed to writing the manuscript. TK and TC helped with data analysis and proofreading of the manuscript. All authors contributed to the article and approved the submitted version.

## Conflict of Interest

The authors declare that the research was conducted in the absence of any commercial or financial relationships that could be construed as a potential conflict of interest.
