# Supplementary material for: Laccase Immobilization Strategies for Application as a Cathode Catalyst in Microbial Fuel Cells for Azo Dye Decolourization
Source: Front Microbiol. 2021 Jan 18;11:620075. doi: 10.3389/fmicb.2020.620075 (PMC7847978; doi:10.3389/fmicb.2020.620075)
Supplement: Supplementary file 1 [file Data_Sheet_1.DOCX]

Laccase immobilization strategies for application as a cathode catalyst in microbial fuel cells for azo dye decolourization

Priyadharshini Mani^1*^, Fidal V T^2^, Taj Keshavarz^1^, Chandra T S^2^ and Godfrey Kyazze^1*^

^1^School of Life Sciences, University of Westminster, London W1W 6UW

^2^ Department of Biotechnology, Indian Institute of Technology (Madras), Chennai-36.

^*^Correspondence: Priyadharshini Mani, Email: [priyadharshu@gmail.com](about:blank); Godfrey Kyazze; G.Kyazze@westminster.ac.uk

Table S1 The performance of the immobilized electrodes in the 2^nd^ cycle of MFC.

| Laccase immobilization methods | Maximum Power Density (mW m^-2^) | Dye decolourization  Efficiency (%) | Relative Enzyme Activity after 2^nd^ cycle (%) |
| --- | --- | --- | --- |
| PANI Lac | 28 | 58 | 61 |
| Nafion Lac | 16.2 | 50 | 40 |
| Cu-Alg Lac | 10 | 65 | 38 |
| Free Lac |  |  |  |

Table S2 The performance of the immobilized electrodes in the 3^rd^ cycle of MFC.

| Laccase immobilization methods | Maximum Power Density (mW m^-2^) | Dye decolourization  Efficiency (%) | Relative Enzyme Activity after 3^rd^ cycle (%) |
| --- | --- | --- | --- |
| PANI Lac | 23.6 | 50 | 45 |
| Nafion Lac | 11 | 35 | 30 |
| Cu-Alg Lac | 6 | 57 | 26 |
| Free Lac |  |  |  |
